# Environmental contaminants and the disproportionate prevalence of type-2 diabetes mellitus among Indigenous Cree women in James Bay Quebec, Canada

**DOI:** 10.1038/s41598-021-03065-6

**Published:** 2021-12-15

**Authors:** Aleksandra Zuk, Eric N. Liberda, Leonard J. S. Tsuji

**Affiliations:** 1 Department of Physical and Environmental Sciences, University of Toronto Scarborough, Toronto, ON Canada; 2 School of Nursing, Faculty of Health Sciences, Queen’s University, 92 Barrie Street, Kingston, ON K7L 3N6 Canada; 3 School of Occupational and Public Health, Ryerson University, Toronto, ON Canada

**Keywords:** Environmental impact, Endocrine system and metabolic diseases

## Abstract

Indigenous populations are disproportionately affected by type 2 diabetes (T2DM) compared to non-Indigenous people. Of importance, the prevalence of T2DM is greater amongst females than males in First Nations communities, in contrast to higher male prevalence reported in non-Indigenous Canadians. Therefore, in this study we extend our previously published work with respect to females, and the potential association between environmental exposures to organochlorine pesticides, such as dichlorodiphenyltrichloroethane (DDT), and dichlorodiphenyldichloroethylene (DDE) to explain the greater prevalence of T2DM among Indigenous females compared to males. Using data from the Multi-Community Environment-and-Health Study, Principal Component Analysis (PCA), examined 9-polychlorinated biphenyl congeners, 7-organic pesticides, and 4-metal/metalloids. Modified Poisson regression with robust error variance estimated adjusted prevalence ratios (PR) and corresponding 95% confidence intervals (95% CI), regressing prevalent T2DM on the newly derived principal components (PC), adjusting for a priori covariates, including parity. We further examined the relationship between high detection concentrations of DDT and tertials of categorized DDE exposures on T2DM among Indigenous Cree women. Among 419 female participants, 23% (n = 95) had physician-diagnosed T2DM. PCA analysis show that DDT and Lead (Pb) loaded highly on the second axis (PC-2), although in opposite directions, indicating the different exposure sources. As previously published, T2DM was significantly associated with PC-2 across adjusted models, however, after further adjusting for parity in this analysis, T2DM was no longer significantly associated with increasing PC-2 scores (PR = 0.88, 95% 0.76, 1.03). Furthermore, we found that the highest detectable levels of DDT, and tertiles of DDE were significantly associated with prevalent T2DM in the fully adjusted model (PR = 1.93, 1.17, 3.19), and (PR = 3.58, 1.10, 11.70), respectively. This cross-sectional analysis suggests organochlorines, specifically, detectable high exposure concentrations of DDT and DDE are associated with prevalent type 2 diabetes, signifying a possible important link between parity and environmental organochlorines pesticides among Indigenous Cree women.

## Introduction

The International Diabetes Federation^1^ reports that 463 million adults (20 to 79 years old) worldwide have diabetes, and another 374 million adults have impaired glucose tolerance, a risk factor in developing diabetes. In Canada, it has been estimated that 3.4 million people have diabetes, with another 5.7 million individuals having prediabetes^2^. Diabetes is caused by an endocrine dysfunction in the homeostatic regulation of blood glucose. Diabetes is classified into the following four general categories. In type 1 diabetes mellitus (T1DM), there is an absolute deficiency in insulin, one of the main hormones responsible for the regulation of blood glucose; however, for type 2 diabetes mellitus (T2DM) there is a relative deficiency in insulin through the progressive loss of insulin secretion from the pancreatic β-cells (i.e., inadequate insulin secretion) and/or increasing insulin peripheral resistance, the bodies inability to respond to insulin^3^. Gestational diabetes mellitus (GDM) may appear in the second or third trimester of pregnancy, when insulin insensitivity is normally increasing^4,5^. Diagnosis of GDM is between 24 and 28 weeks of gestation among women not previously found to have diabetes^3^. Screening for and diagnosis of GDM is accomplished using either a “one-step” (recommended by International Association of Diabetes and Pregnancy Study Groups^6^ or an older “two-step” (non-fasting) approach recommended by the American College of Obstructions and Gynecologists. Currently, as cited by the American Diabetes Association (2021), the two approaches to diagnosis GDM have yielded inconsistent population-wide outcomes and a uniform approach is yet to be established^3^. Lastly, there are other specific (less common) types of diabetes, such as, monogenic diabetes syndrome (rare beta cell function genetic defects), exocrine pancreatic diseases (complications of certain diseases), and drug- or chemical-induced diabetes^3^.

Globally, approximately 90% to 95% of all cases of diabetes are T2DM^7^, and the prevalence of T2DM has been increasing^8^. The etiology of type 2 diabetes is not known completely, however, the cause of this metabolic disorder characterized by progressive loss of beta-cell function leading to hyperglycemia is multifactorial, including genetics, environmental, and health-related factors. For instance, factors such as genes and family history of diabetes, increasing age, racial and ethnic background disparities, physical inactivity, overweight or obesity (body mass index 25–29.9 kg/m^2^, or > 30 kg/m^2^, respectively) leading to inflammation and immune dysregulation, and less defined, the role of environmental pollutants (e.g., organic pollutants or toxic metals). Despite the numerous pathophysiological studies that have contributed to our understanding of diabetes mellitus^9^, more hypothesis-driven research is needed to determine relationships between environmental factors and differing pathophysiological processes that underlie type 2 diabetes^10^.

Worldwide, the age-standardized prevalence of T2DM has been reported to be several times higher in Indigenous populations than their non-Indigenous counterparts^11,12^. In Canada, Indigenous peoples (i.e. First Nations, Metis, and Inuit as defined by the Canadian *Constitution Act 1982*) are disproportionately living with higher rates of T2DM, but there is a relative paucity of information related to Metis and Inuit peoples compared to First Nations people^13,14^. On-reserve First Nations residents have the highest age-adjusted prevalence for T2DM being more than threefold higher than Canadian non-Indigenous population, 17.2% compared to 5%, respectively^13,14^. In addition, Canadian Indigenous peoples bear a disproportionate burden of the complications associated with T2DM compared to non-Indigenous Canadians, being hospitalized more often with diabetes-related conditions, and die more often from these complications than their non-Indigenous Canadian counterparts^13–16^.

In 2014, estimated worldwide age-adjusted prevalence for T2DM was 9.0% for men and 7.9% for women^17^; this difference was also noted in 1980 (8.3% men and 7.5% women)^18^. American Diabetes Association^5^ lists being male as a risk factor in their Diabetes Risk Test. Of importance to the present study, the prevalence of T2DM in First Nations communities is greater in females than males, sharply contrasting observations in non-Indigenous Canadian population^13,16^. Age-standardized T2DM prevalence rates are reported to be higher for First Nations women (over 20%), than, in contrast to the ~ 16% for First Nations men^15^. In the James Bay Cree First Nations of northern Quebec, Canada, the crude prevalence were found to be higher in women than men for data collected in 1989^19^. More recently, for the same population Dannenbaum et al.^20^ reported that more Cree women are living with diabetes than men, 62.2% compared to 37.8%, respectively. The reason behind why Cree women are disproportionally affected by diabetes is yet to be elucidated, however, Dannenbaum et al.^20^ suggested the cause could be multifactorial with factors, such as physical activity and obesity, including excessive weight gain during pregnancy being of importance. Crowshoe et al.^21^ and Halseth et al.^22^ suggests that the disproportionate burden of T2DM affecting First Nations women may be related to the higher prevalence of GDM in First Nations women compared to non-Indigenous women. Indeed, Indigenous women in Canada experience GDM rates 2 to 3 times higher than non-Indigenous women^15^.

We agree with Dannenbaum et al.^20^ that the issue whereby First Nation women have greater prevalence of T2DM than their male counterparts is multifactorial—and like Crowshoe et al.^21^ and Halseth^22^, GDM is critical to the development of T2DM—but we also hypothesize that lipophilic contaminants may be playing a role. Local sources and long-range transport of contaminants have been identified as sources of contaminants for First Nations people in Canada, through direct exposure and/or indirect exposure through consumption of wild game and fish^23,24^.

In reviews of contaminants and their association with T2DM by Taylor et al.^25^ and Kuo et al.^26^, it was reported that results were suggestive that relationships existed between persistent organic pollutants (e.g. dichloro-diphenyl-dichloroethylene, DDE) and T2DM. Further, in a study by Pal et al.^27^ of two First Nations in northern Ontario, Canada, evidence was presented that persistent organic pollutants (e.g. DDE) were higher in T2DM individuals compared to non-T2DM people. In Canada, Indigenous people are disproportionately affected by obesity^28^. The environmental obesogens hypothesis has been proposed as contributing to obesity through endocrine hormone mimicry^29,30^; however, in a cross-sectional study among Indigenous First Nation adults, Akbar et al.^31^ show no obesogenic association between persistent organic pollutants (i.e., polychlorinated biphenyls and organochlorine pesticides) and measures of morphometry, including body mass index. And, although DDT and its DDE metabolite may have endocrine disruption actions (e.g., androgen receptor antagonist), the specific mechanism by which these contaminants function have yet to be elucidated^32^—especially as it relates to T2DM.

Recently, Zuk et al.^33^ examined the association of complex environmental contaminant mixtures and T2DM in First Nation Cree community members of northern Quebec, Canada, using Principal Component Analysis to reduce the dimensionality of the concentration data for 20 contaminants to orthogonal principal component axes and found an association between DDT and T2DM^33^. In the present study, we will extend the work of Zuk et al.^33^ with respect to only females, and the potential association between DDT and DDE with T2DM.

## Methods

### Data sources

**Figure 1:**
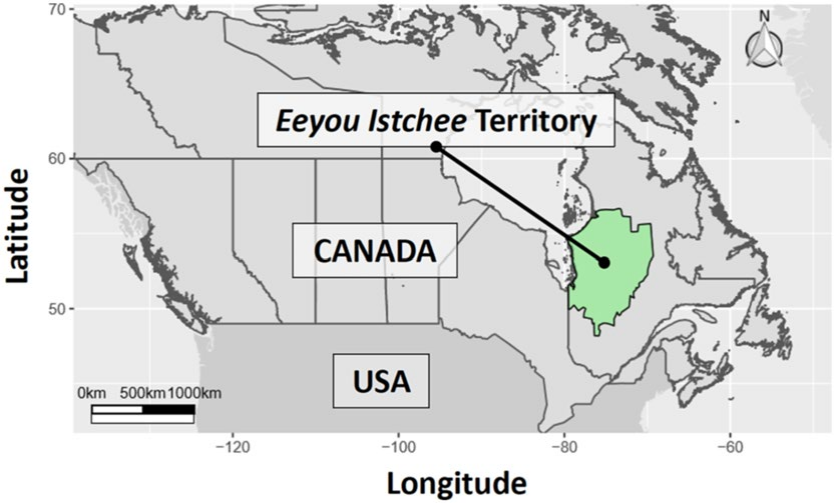
Map of the study region in northern Quebec, Canada.

The Cree First Nation (Indigenous) communities of the *Eeyou Istchee* territory are located on the eastern side of the James Bay in Quebec, Canada (Fig. 1). The *Nituuchischaayihtitaau Aschii*: Multi-Community Environment and-Health Study was conducted in seven communities from 2005 to 2009, with two communities having been studied previously (2002). The aim of the *Nituuchischaayihtitaau Aschii* study was to provide health assessment and investigate the effect of lifestyle, environmental contaminant exposure, and diet among the participants from the *Eeyou Istchee* territory. Participants underwent physical examinations, completed health surveys, and provided blood samples, among other tissues, for analysis. The data and sample collection was conducted by experienced field research nurses who also conducted a medical chart review to verify the self-reported survey information related to disease status.

### Study population

Of the initial 1750 recruited participants, we assessed adult females over the age of 20 years old who had medical-chart verified T2DM diagnoses, complete environmental contaminant body burden profiles, underwent physical examination, completed the health survey, and provided blood for analyses. We excluded those who had T1DM diagnoses. In total, 419 women representing seven of nine communities from the *Eeyou Istchee* territory were carried forward for analyses.

### Environmental contaminant analyses

We have previously published the analytical methods, including the limits of detection, for both organic and metal and metalloid contaminants^33–35^. Briefly, organic contaminants were assessed in blood plasma post solid-phase extraction and florisil column cleaning prior to measurement on an Agilent high resolution gas chromatography-mass spectrometry (HRGC-MS; Agilent 6890 and Agilent 5973) unit. The analytes of organic contaminants in blood plasma included polychlorinated biphenyl (PCBs) congeners (CBs 99, 187, 183, 180, 170, 153, 128, 118, and 105) and organic pesticides (cis-Nonachlor, Dichlorodiphenyltrichloroethane [p,p′-DDT], Dichlorodiphenyldichloroethylene [p,p′-DDE], Hexachlorobenzene [HCB], Mirex, oxy-chlordane, trans-Nonachlor. Whole blood samples were assessed for Lead (Pb), total mercury (Hg), cadmium (Cd), and selenium (Se) using a Perkin Elmer Sciex Elan 6000 inductively coupled plasma-mass spectrometer (ICP-MS). All environmental contaminant analyses were conducted at the Institut National de Santé Publique du Québec (INSPQ), the reference laboratory for the Arctic Monitoring and Assessment Program.

### Risk factor covariates

Demographic information such as sex and age, and behavioral risk factors such as smoking status, were obtained from the self-reported surveys. As previously described^33^, age was transformed into three categories as follows: 20–39, 40–59, and ≥ 60 years of age. Self-reported education was categorized as: completed less than high school, completed high school, and completed some or more post-secondary schooling. Smoking status was defined as current or former and never smoker. Fasting blood samples were drawn by clinical field nurses who also took anthropometric measures such as standing height (cm), weight (kg) for body mass index (BMI). Total lipids were determined as described by Rylander et al.^36^. Parity was determined by developing a composite variable of the two questions: “How many children did you give birth to?” and “How many pregnancies did you have that resulted in a miscarriage?”.

### Statistical methods

We performed a scaled and centered principal component analysis (PCA) to reduce the 21 contaminant variables into a smaller subset of uncorrelated (orthogonal) predictor variables^37,38^. The derived principal components scores (PCs) with eigenvalues exceeding 1.0 were used as independent predictors in the regression analysis of T2DM. Prior to PCA, the contaminant concentrations were log_10_ (variate + 1) transformed in order to improve the distribution of these variables. Component loadings that had absolute scores of 0.5 or greater were considered important for the given principal component axis.

Descriptive statistics of the variables were stratified by T2DM status and are reported as means ± standard deviation, or frequencies and percentages, were appropriate. Modified Poisson regression with robust error variance was used to calculate adjusted prevalence ratios (PR) and their corresponding 95% confidence intervals (95% CI) using SAS PROC GENMOD^39,40^. Multivariable models were used to assess the association between T2DM and principal components, while adjusting for the following a priori covariates: age, lipid concentrations, BMI, smoking status, education, and parity.

Based on the significant associations obtained from the regression of PCA axes on prevalent T2DM, we further explored the role of p,p′-DDT and p,p′-DDE using a subsequent sensitivity analysis. Owing to the low numbers of DDT exposed participants, we categorized this variable into two levels; the top 10% of exposures and compared them to those that were below the limit of detection (90%). We also categorized DDE exposures into tertials (< 25% [reference group], 25–75%, and ≥ 75% of concentrations. Both DDT and DDE models were adjusted for age, lipids, BMI, smoking status, education, and parity. As above, a modified Poisson regression with robust error variance was used to calculate adjusted prevalence ratios (PR) and their corresponding 95% confidence intervals (95% CI).

All statistical analyses were conducted using SAS v9.4 (SAS Institute, Inc., Cary, NC) and the geographic map and PCA loadings figures were generated using R (version 3.5.3, Vienna, Austria).

### Ethics approval and consent to participate

The *Nituuchischaayihtitaau Aschii*—Multi-Community Environment-and-Health Study was conducted in accordance with relevant guidelines, regulations, and research agreements. Informed consent was obtained from all participants or their guardians in Cree, French, or English languages. The study was approved by the ethics board of McGill and Laval Universities in partnership with the Cree Board of Health and Social Services of James Bay and McMaster University.

## Results

### Descriptive results

Demographic, risk factor, and contaminant concentration summary statistics are presented in Table 1 (part of this Table is reproduced from Zuk et al.^33^). Of the 419 female participants, 95 (23%) presented with a T2DM diagnosis and 324 (77%) did not. The mean age was 47.9 (± 14.7) years old for those with a T2DM diagnosis and 38.5 (± 13.9) years for those not being diagnosed with T2DM. Among respondents diagnosed with T2DM, the mean BMI, current smoking status, parity, and total lipids was 39 kg/m^2^, 30.8%, 5.4 pregnancies, and 6.4 g/L, respectively. For those without T2DM, the mean BMI was 34.5 kg/m^2^, 54.7% were current smokers, the mean number of pregnancies was 4.1, and the mean total lipids were 5.8 g/L. The mean concentrations of contaminants for participants was varied, with the highest organic concentration of 2.96 µg/L being found for p,p′-DDE in those diagnosed with T2DM.

**Table 1:** Female characteristics stratified by type 2 diabetes mellitus status: results from the *Nituuchischaayihtitaau Aschii*—Multi-Community Environment-and-Health Study (2005–2009).

Characteristics		Total population (n = 419)	Type 2 diabetes status	
			Present	Absent
			N (%); or mean ± SD	N (%); or mean ± SD
Demographic	Participants	419	95 (23%)	324 (77%)
	Age (years)		47.9 ± 14.7	38.5 (± 13.9)
	**Education**			
	Less than high school	412	34 (37.4%)	55 (17.1%)
	Some or completed high school		35 (38.5%)	181 (56.4%)
	Some or completed college or higher (R)		22 (24.2%)	85 (26.5%)
Risk factors	BMI (kg/m^2^)	411	39 ± 8.6	34.5 ± 6.5
	Smoking status, current/occasional smoker compared to former/never (R)	413	28 (30.8%)	176 (54.7%)
	Mean number of pregnancies (Parity)	330	5.4 ± 3.3	4.1 ± 2.4
	Total lipids (g/L)^a^	419	6.4 ± 1.6	5.8 ± 1.1
Contaminants (µg/L)^b^	**PCBs**			
	PCB 99		0.116 ± 4.061	0.044 ± 3.470
	PCB 105		0.058 ± 3.331	0.025 ± 2.563
	PCB 118		0.262 ± 4.583	0.074 ± 4.508
	PCB 128		0.019 ± 1.639	0.015 ± 1.331
	PCB 138		0.565 ± 4.641	0.175 ± 5.077
	PCB 153		1.204 ± 4.877	0.376 ± 5.586
	PCB 170		0.259 ± 4.592	0.093 ± 4.751
	PCB 180		0.887 ± 5.069	0.290 ± 5.677
	PCB 183		0.112 ± 3.919	0.044 ± 3.442
	PCB 187		0.348 ± 4.863	0.117 ± 5.077
	**Organochlorines**			
	cis-Nonachlor		0.052 ± 2.945	0.024 ± 2.405
	p,p′-DDE		2.958 ± 3.305	1.043 ± 3.746
	p,p′-DDT		0.035 ± 1.811	0.027 ± 1.326
	Hexachlorobenzene (HCB)		0.120 ± 3.054	0.055 ± 2.878
	Mirex		0.161 ± 4.620	0.062 ± 4.432
	oxy-Chlordane		0.085 ± 3.249	0.035 ± 2.862
	trans-Nonachlor		0.150 ± 3.527	0.052 ± 3.510
	**Metals and metalloids**			
	Cadmium, Cd (nmol/L)		5.563 ± 2.729	8.849 ± 2.782
	Total mercury, Hg (nmol/L)		28.275 ± 3.798	14.862 ± 3.809
	Lead, Pb (µmol/L)		0.131 ± 2.848	0.119 ± 3.003
	Selenium, Se (µmol/L)		2.209 ± 1.218	2.118 ± 1.157

Missing values among adult females: Education (n = 7, 1.7%); BMI (n = 8, 1.9%); Smoking status (n = 6, 1.4%).

N, frequency value; %, percentage; BMI, Body mass index; R, reference category; PCB, Polychlorinated biphenyl congeners; *p,p′-*DDT, Dichlorodiphenyltrichloroethane; *p,p′-*DDT, Dichlorodiphenyldichloroethylene.

Part of this Table is reproduced from Zuk et al.^33^.

^a^Lipid concentrations were determined using methods described by Rylander et al. 2012.

^b^Presented are geometric mean ± standard deviation (SD).

### Principal component analysis (PCA) loadings

**Figure 2:**
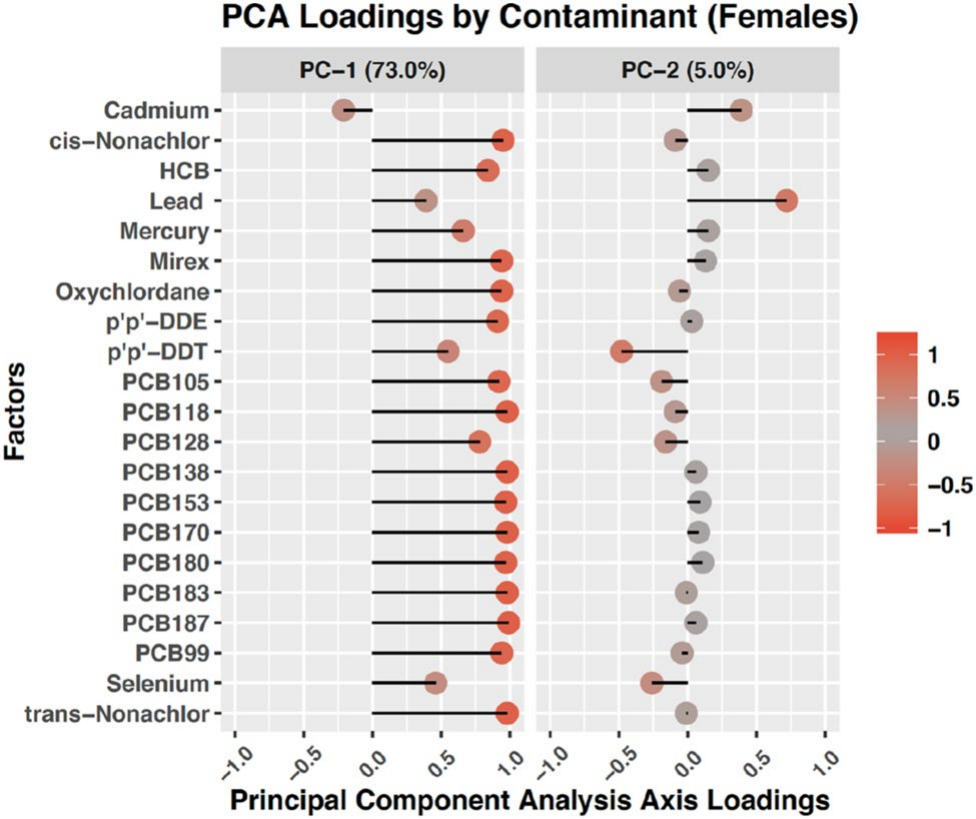
Principal component (PC) loadings of contaminants. Part of this Figure is reproduced from Zuk et al.^33^.

The PC loadings for the participants in this study are shown in Fig. 2 (part of this Figure is reproduced from Zuk et al.^33^). The first PC axis explained 73% of the total sample variance and was highly and positively loaded for organic contaminants such as PCBs and organochlorines and mercury. The second PC axis explained 5% of the sample variation and was highly and negatively loaded for DDT, and positively loaded for Pb. Combined, these two PC axes represented 78% of the total sample variation in the original contaminant concentrations.

### PCA associations with type 2 diabetes

The results of the multivariable modified Poisson regression analyses are presented in Table 2 (part of this Table is reproduced from Zuk et al.^33^). This analysis assessed the association between prevalent T2DM and the computed orthogonal PCs of the environmental contaminant mixture. In the unadjusted model, PC-1 (Organic contaminants and mercury) and PC-2 (negative loading for DDT and positive loading for Pb) both had significant associations with T2DM (PR = 1.34, 95% CI = 1.18–1.52; PR = 0.83, 95% CI = 0.73–0.95, respectively). After adjusting for age, lipids, BMI, smoking status, and education, Model 1 retained its significant association with PC-2 (PR = 0.84, 95% CI = 0.72–0.98), but lost significance with PC-1. The final model (Model 2), which added parity as a confounder, resulted in no significant associations for any PC axis with T2DM.

**Table 2:** Multivariable adjusted prevalence ratios (95% Confidence Intervals) for prevalent type 2 diabetes mellitus and principal component axes among adult females over 20 years of age using data from the *Nituuchischaayihtitaau Aschii*—Multi-Community Environment-and-Health Study (2005–2009).

Models	PR	95% confidence interval		P value*
		Lower limit	Upper limit	
**Unadjusted**				
PC1	1.34	1.18	1.52	** < 0.0001**
PC2	0.83	0.73	0.95	**0.0053**
**Model 1**				
PC1	1.07	0.84	1.36	0.6009
PC2	0.84	0.72	0.98	**0.0220**
**Model 2**				
PC1	1.07	0.81	1.41	0.6299
PC2	0.88	0.76	1.03	0.1080

Part of this Table is reproduced from Zuk et al.^33^.

PC1 and PC2, first and second orthogonal principal component axes, respectively; PR, prevalence ratio. *Significance (p-value < 0.05). Model 1: adjusted for age, lipids, BMI, smoking status, education; Model 2: Model 1 + parity.

Significant values are in bold (p-value < 0.05).

### DDT and DDE associations with type 2 diabetes

Based on the association and direction with PC-2 with prevalent T2DM in Model 1, and noting the effect of parity on Model 2, we further explored the role of DDT and DDE may play in this association (Table 3). The unadjusted model showed a significant association between DDT and prevalent T2DM (PR = 3.34, 95% CI 2.30, 4.83). In the fully adjusted model, after adjusting for age, lipids, BMI, smoking status, education, and parity, the strength of association was attenuated but remained significantly associated with T2DM (PR = 1.93, CI = 1.17, 3.19). Similarly, the unadjusted model of DDE was shown to have a significant association with prevalent T2DM in both the < 25% vs. 25–75% tertial group, and the < 25% vs. ≥ 75% tertial groups. After full adjustment (as above), this significant observation remained, with the < 25% vs. 25–75% tertial group having a PR of 2.7 (95% CI = 0.98, 7.47), and the < 25% vs. ≥ 75% tertial group having a PR of 3.58 (95% CI = 1.10, 11.70).

**Table 3:** Multivariable adjusted prevalence ratios (95% Confidence Intervals) for prevalent type 2 diabetes mellitus and p,p′-DDT (Dichlorodiphenyltrichloroethane) and p,p′-DDE (Dichlorodiphenyldichloroethylene) among adult females over 20 years of age using data from the *Nituuchischaayihtitaau Aschii*—Multi-Community Environment-and-Health Study (2005–2009).

Models	PR	95% confidence interval		P value*
		Lower limit	Upper limit	
**Unadjusted**				
p,p′-DDT^a^	3.34	2.30	4.83	** < 0.0001**
p,p′-DDE^b^	4.07	1.50	11.08	**0.0060**
p,p′-DDE^c^	8.19	3.05	22.04	** < 0.0001**
**Full model**				
p,p′-DDT^a^	1.93	1.17	3.19	**0.0098**
p,p′-DDE^b^	2.70	0.98	7.47	0.0550
p,p′-DDE^c^	3.58	1.10	11.70	**0.0348**

PR, prevalence ratio. *Significant values are in bold (p-value < 0.05). Full Model: adjusted for age, lipids, BMI, smoking status, education, parity.

Significant values are in bold.

^a^ ≥ 10% detectable exposure group compared to below the limit of detection.

^b^25–75% tertial exposure group compared to < 25% tertial exposure group.

^c^ ≥ 75% tertial exposure group compared to < 25% tertial exposure group.

## Discussion

This cross-sectional analysis revealed that a positive DDT loading on the second principal component (PC-2) was associated with T2DM with decreasing axis score (i.e., PR greater than 1), the association was no longer significant once we accounted for parity. This suggests that parity impacts the association between DDT concentration and T2DM status. Subsequent analysis comparing those exposed to unexposed DDT groups had a strong positive significant association with prevalent T2DM in the fully adjusted model. Similarly, in the exposed third tertile compared to the unexposed first tertile DDE groups, the strength of effect was stronger with the prevalent T2DM in the exposed DDE group. However, when examining the second DDE tertile compared to the unexposed DDE group the strength of association with T2DM was only slightly attenuated, and borderline significant. Nonetheless, taken together these results are suggestive that parity influences the concentration of contaminants (i.e. DDT and DDE) in Cree women that is impacting the prevalence of T2DM.

Glucose metabolism during pregnancy differs substantially from the non-pregnant state, as a metabolic change is required to meet the energy needs of both mother and growing fetus^4,41,42^. This maternal shift in glucose metabolism is highlighted by increasing insulin insensitivity and mediated through a complex series of interactions between placental and maternal hormones^4,43^. Although the exact mechanism for insulin insensitivity during pregnancy is not completely known^44^ what is known is that insulin insensitivity is orchestrated through a series of hormonal changes during the gestation period^4,41,42^.

Pregnancy stresses the body by producing on hormonal, physiological, metabolic, and lifestyle changes, which have long-term health effects for women. The relationship between parity and type 2 diabetes has been studied in various populations, however, research is conflicting. In a cross-sectional study, the number of live births (parity) in a group of Hispanic postmenopausal women from Columbia showed an association with parity and diabetes even after adjusting for age, body mass index, and family history among multiparous women when compared to the referent nulliparous group. The magnitude of effect was strongest (fivefold odds ratio) for women (≥ 6 number of births) although data presented wide confidence intervals^45^. Similarly, data from the Dongfeng-Tongji cohort study show cross-sectionally a significant increasing trend in type 2 diabetes risk among women who had two, three, and four or more live births^46^. More recently, Shi et al.^47^ in a cross-sectional analysis of normal-weight undiagnosed type 2 diabetes postmenopausal women report that parity was not significantly associated with increased risk of metabolic syndrome. However, the odds of metabolic risk factor such abdominal obesity was significantly associated with multiparity in normal weight Chinese women^47^.

In the Singapore Chinese Health Study, a prospective cohort, found that older women (45–74 years of age) who were free of diabetes at baseline, including other cardiovascular comorbidities report a positive graded hazard ratio association measure with parity and self-reported type 2 diabetes that was diagnosed by a physician^48^. Similarly, population-based prospective Atherosclerosis Risk in Communities study showed that after adjusting for sociodemographic, clinical, and lifestyle factors, the grandmultiparity (five or more births) cox proportional hazard regression model was significantly associated with increased diabetes risk^49^. Furthermore, data from the large population-based cohort, the Risk Evaluation of Cancers in Chinese Diabetic Individuals (REACTION) study examined cross-sectionally the association between parity and risk of maternal diabetes in females (≥ 40 years of age). Huo et al.^50^ reported a higher odds of diabetes among multiparous and nulliparious women when compared to primiparous women.

Channa et al.^51^ investigated body burdens of DDT in maternal plasma at delivery in three Indian Ocean coastal regions of KwaZulu-Natal Province, South Africa, who continue to spray as part of the Malaria Vector Control Program. These authors found that parity was associated with p,p′-DDE, and p,p′-DDT concentrations (ng/g lipids) among women with two or more children. In Vietnam, researchers found that p,p′-DDT concentrations in maternal milk was higher in multiparas than those in primiparas mothers^52^. However, an Australian longitudinal study of pesticide residue in human breastmilk, found no association between *p,p*′-DDE concentrations and parity^53^. Conversely, in India, a risk assessment study showed higher mean concentrations of p,p′-DDE in primiparae than in multiparae samples of human breastmilk. Furthermore, the mean concentration of summed DDTs were also found to be higher in primiparae than the multiparae group although the difference was non-significant due to the small sample size with only fifty-three human breast milk samples among women admitted to a hospital maternity ward^54^. Maternal parity differences likely contributed to the excretion route of in breastmilk (i.e., breastfeeding or lactation)^52^.

Taking everything into account, it is understandable that pregnancy has been referred to as a physiological stress test of the pancreatic β-cells, which must compensate for the normal increase in insulin resistance, by increasing the secretion of insulin, to maintain glucose homeostasis^55^. If the β-cells have a defect and cannot respond appropriately to the insulin insensitivity challenge, GDM will be the result, and perhaps T2DM post-pregnancy^55^. Worldwide, the prevalence of GDM ranges from 0.8% in Nepal to 51% in Saudi Arabia^56^. In Canada, the prevalence rate has been estimated to be between 3 and 20% dependent on risk factors^57^. For Indigenous women in Canada, GDM is experienced at rates of 2 to 3 times higher than that of non-Indigenous women^15^. Moreover, women who have had GDM, face a greater risk of developing T2DM^41^.

Thus, if First Nation women in Canada or in our case First Nation Cree women of Quebec were exposed to an additional stressor during pregnancy, that added to the insulin insensitivity already present, this may explain the disproportionate number of Cree women with T2DM compared to Cree men with T2DM. However, it would also have to be shown that non-Indigenous women would not be exposed to the same stressor, because the prevalence of T2DM in women of the non-Indigenous Canadian population is less than the prevalence of T2DM in their male counterparts^13,16^. The pregnancy stressor we put forward is DDT and DDE, and other lipophilic organochlorines may also be of concern.

First off, it is well known that Indigenous people worldwide typically have higher body burdens of lipophilic contaminants (e.g. organochlorines) compared to their non-Indigenous counterparts, and this is well known for the Cree of James Bay, Canada^34,58,59^. Moreover, Cree women have relatively high concentrations of lipophilic contaminants even when compared to other Indigenous women^34,58,59^, and non-Indigenous pregnant Canadian women in the 3rd trimester^60^. Second, it is known that during pregnancy that there is an energy production switch from carbohydrates to lipids^41,42^; thus, lipolysis should result in the release of lipids into the maternal circulation and concomitantly an increase in lipophilic contaminants on a wet weight basis. Indeed, it is well established that lipids increase from the 1st trimester through the 2nd and peaks in the 3rd trimester at delivery^61,62^. Likewise, mirroring the lipid trend, organochlorine concentrations on a wet weight basis, have been shown to increase from the 1st to 3rd trimesters in several studies^63–67^. However, this organochlorine wet weight positive trend during pregnancy is not apparent when organochlorine concentrations are lipid adjusted (see for e.g. Longnecker et al.^68^; Hansen et al.^69^; Knudsen et al.^62^). This is one of the reasons why there has been some controversy about when the best time to sample for organochlorines during pregnancy, that is, does a critical window exist for sampling, because of the dynamic nature of organochlorine concentrations during pregnancy^70–72^. It should be emphasized that wet weight concentrations are what is actually measured analytical on a gram/L (or equivalent) basis; wet weight is more closely equivalent to a dose in a pharmacologic sense. When health endpoints are being examined, changes in dosage are of importance. In contrast, lipid-adjusted concentrations of contaminants are imputed numbers used to describe body burdens, typically in equilibrium, not dose.

If we consider the increase of organochlorines concentrations (wet weight) from the 1st to the 3^rd^ trimester as an added stressor, to the already stressed maternal glucose metabolic system—taking into account that organochlorines have been linked to increased insulin resistance (e.g. Ngwa et al.^73^)—then β-cells already pushed to their physiological limit trying to compensate (for normal increased insulin insensitivity during pregnancy) by producing more insulin, may be irreparable impacted. Under this scenario, we would predict that relatively unexposed (to organochlorines) pregnant women would not be impacted by the added stressor of increasing organochlorine concentrations during pregnancy, because the contaminants would not be present or only at very low concentrations; thus, T2DM would be less prevalent in this group compared to an exposed group. This is in fact what we found among the Cree women of the present study. So, it is not parity per se that was associated with prevalence of T2DM, but parity and a relatively high body burden of organochlorines.

Limitations in the present study include the cross-sectional nature of the data analyzed, thus, not allowing the establishment of causality. Further, there are many other factors during pregnancy that could impact insulin resistance. The sex of the fetus may be of importance^74^, in that female fetuses have been associated with greater maternal insulin resistance^75^, and a slightly higher future risk of T2DM^55^. Inheritance factors may also be of importance^41^, with eight different genetic loci (i.e. TCF7L2, GCK, KCNJ11, KCNQ1, CDKAL1, IGF2BP2, MTNR1B and IRS1) being associated with both increased risk of GDM and T2DM^42^. The Multi-Community Environment and Health study also did not collect historical data related to puberty or age at menarche and therefore, supplemental mediated pathways could not additionally be examined in relation to T2DM. Lastly, the body’s microbiome could also be a contributor to insulin resistance^42^.

In conclusion, type 2 diabetes disproportionately burdens Indigenous Cree women. In this cross-sectional study we show that organochlorines, specifically, the highest exposure concentrations of p,p′-DDT and its primary metabolite p,p′-DDE were associated with prevalent type 2 diabetes, suggesting a link between parity and these environmental organochlorines pesticides.
